# A fully automatic gridding method for cDNA microarray images

**DOI:** 10.1186/1471-2105-12-113

**Published:** 2011-04-21

**Authors:** Luis Rueda, Iman Rezaeian

**Affiliations:** 1 School of Computer Science, University of Windsor 401 Sunset Avenue, Windsor, Ontario, N9B 3P4, Canada

## Abstract

**Background:**

Processing cDNA microarray images is a crucial step in gene expression analysis, since any errors in early stages affect subsequent steps, leading to possibly erroneous biological conclusions. When processing the underlying images, accurately separating the sub-grids and spots is extremely important for subsequent steps that include segmentation, quantification, normalization and clustering.

**Results:**

We propose a parameterless and fully automatic approach that first detects the sub-grids given the entire microarray image, and then detects the locations of the spots in each sub-grid. The approach, first, detects and corrects rotations in the images by applying an affine transformation, followed by a polynomial-time optimal multi-level thresholding algorithm used to find the positions of the sub-grids in the image and the positions of the spots in each sub-grid. Additionally, a new validity index is proposed in order to find the correct number of sub-grids in the image, and the correct number of spots in each sub-grid. Moreover, a refinement procedure is used to correct possible misalignments and increase the accuracy of the method.

**Conclusions:**

Extensive experiments on real-life microarray images and a comparison to other methods show that the proposed method performs these tasks fully automatically and with a very high degree of accuracy. Moreover, unlike previous methods, the proposed approach can be used in various type of microarray images with different resolutions and spot sizes and does not need any parameter to be adjusted.

## Background

Microarrays are one of the most important technologies used in molecular biology to massively explore how the genes express themselves into proteins and other molecular machines responsible for the different functions in an organism. These expressions are monitored in cells and organisms under specific conditions, and have many applications in medical diagnosis, pharmacology, disease treatment, just to mention a few. We consider cDNA microarrays which are produced on a chip (slide) by hybridizing sample DNA on the slide, typically in two channels. Scanning the slides at a very high resolution produces images composed of sub-grids of spots. Image processing and analysis are two important aspects of microarrays, since the aim of the whole experimental procedure is to obtain meaningful biological conclusions, which depends on the accuracy of the different stages, mainly those at the beginning of the process. The first task in the sequence is gridding [1-5], which if done correctly, substantially improves the efficiency of the subsequent tasks that include segmentation [6], quantification, normalization and data mining. When producing cDNA microarrays, many parameters are specified, such as the number and size of spots, number of sub-grids, and even their exact locations. However, many physicochemical factors produce noise, misalignment, and even deformations in the sub-grid template that it is virtually impossible to know the exact location of the spots after scanning, at least with the current technology, without performing complex procedures. Roughly speaking, gridding consists of determining the spot locations in a microarray image (typically, in a sub-grid). The gridding process requires the knowledge of the sub-girds in advance in order to proceed (sub-gridding).

Many approaches have been proposed for sub-gridding and spot detection. The Markov random field (MRF) is a well known approach that applies different constraints and heuristic criteria [1,7]. Mathematical morphology is a technique used for analysis and processing geometric structures, based on set theory, topology, and random functions. It helps remove peaks and ridges from the topological surface of the images, and has been used for gridding the microarray images [8]. Jain's [9], Katzer's [10], and Stienfath's [11] models are integrated systems for microarray gridding and quantitative analysis. A method for detecting spot locations based on a Bayesian model has been recently proposed, and uses a deformable template to fit the grid of spots using a posterior probability model for which the parameters are learned by means of a simulated-annealing-based algorithm [1,3]. Another method for finding spot locations uses a hill-climbing approach to maximize the energy, seen as the intensities of the spots, which are fit to different probabilistic models [5]. Fitting the image to a mixture of Gaussians is another technique that has been applied to gridding microarray images by considering radial and perspective distortions [4]. A Radon-transform-based method that separates the sub-grids in a cDNA microarray image has been proposed in [12]. That method applies the Radon transform to find possible rotations of the image and then finds the sub-grids by smoothing the row or column sums of pixel intensities; however, that method does not automatically find the correct number of sub-grids, and the process is subject to data-dependent parameters.

Another approach for cDNA microarray gridding is a gridding method that performs a series of steps including rotation detection and compares the row or column sums of the top-most and bottom-most parts of the image [13,14]. This method, which detects rotation angles with respect to one of the axes, either *x *or *y*, has not been tested on images having regions with high noise (e.g., the bottom-most  of the image is quite noisy).

Another method for gridding cDNA microarray images uses an evolutionary algorithm to separate sub-grids and detect the positions of the spots [15]. The approach is based on a genetic algorithm that discovers parallel and equidistant line segments, which constitute the grid structure. Thereafter, a refinement procedure is applied to further improve the existing grid structure, by slightly modifying the line segments. Using maximum margin is another method for automatic gridding of cDNA microarray images based on maximizing the margin between rows and columns of spots [16]. Initially, a set of grid lines is placed on the image in order to separate each pair of consecutive rows and columns of the selected spots. Then, the optimal positions of the lines are obtained by maximizing the margin between these rows and columns using a maximum margin linear classifier. For this, a SVM-based gridding method was used in [17]. In that method, the positions of the spots on a cDNA microarray image are first detected using image analysis operations. A set of soft-margin linear SVM classifiers is used to find the optimal layout of the grid lines in the image. Each grid line corresponds to the separating line produced by one of the SVM classifiers, which maximizes the margin between two consecutive rows or columns of spots.

## Results and Discussion

For testing the proposed method (called Optimal Multi-level Thresholding Gridding or OMTG), three different kinds of cDNA microarray images have been used. The images have been selected from different sources, and have different scanning resolutions, in order to study the flexibility of the proposed method to detect sub-grids and spots with different sizes and features.

The first test suite consists of a set of images drawn from the Stanford Microarray Database (SMD), and corresponds to a study of the global transcriptional factors for hormone treatment of *Arabidopsis thaliana *samples. The images can be downloaded from smd.stanford.edu, by selecting "Hormone treatment" as category and "Transcription factors" as subcategory. Ten images were selected, which correspond to channels 1 and 2 for experiments IDs 20385, 20387, 20391, 20392 and 20395. The images have been named using AT (which stands for *Arabidopsis thaliana*), followed by the experiment ID and the channel number (1 or 2).

The second test suite consists of a set of images from Gene Expression Omnibus (GEO) and corresponds to an Atlantic salmon head kidney study. The images can be downloaded from ncbi.nlm.nih.gov, by selecting "GEO Datasets" as category and searching the name of the image. Eight images were selected, which correspond to channels 1 and 2 for experiments IDs GSM16101, GSM16389 and GSM16391, and also channel 1 of GSM15898 and channel 2 of GSM15898. The images have been named using GSM followed by the experiment ID, and the channel number (1 or 2).

The third test suite consists of two images, obtained from a dilution experiment (DILN) and correspond to channels experiments IDs Diln4-3.3942.01A and Diln4-3.3942.01B [18]. The specifications of the cDNA microarray images for each of these three test suites are summarized in Table 1.

**Table 1:** Test suite used to evaluate the performance of the methods

Suite Name	SMD	GEO	DILN
Database Name	Stanford Microarray Database	Gene Expression Omnibus	Dilution Experiment

Image Format	Tiff	Tiff	Tiff

No. of Images	10	8	2

Image Resolution	1910 × 5550	1900 × 5500	600 × 2300

Sub-grid Layout	12 × 4	12 × 4	5 × 2

Spot Layout	18 × 18	13 × 14	8 × 8

Spot Resolution	24 × 24	12 × 12	from 12 × 12 to 3 × 3

The specifications of the three datasets of cDNA microarray images used to evaluate the proposed method.

To assess the performance of the proposed method, we consider the percentage of the grid lines that separate sub-grids/spots incorrectly, marginally and perfectly. These quantities were found by visually analyzing the result of the gridding produced by our method. For SMD and GEO, our gridding was not compared with the gridding currently available in these databases. For DILN, apart from the visual analysis, we also apply segmentation and quantification by computing the volume of log of intensity and relate these to the rate of dilution in the biological experiment. For the implementation, we used Matlab2010 on a Windows 7 platform and an Intel core i7 870 cpu with 8GB of memory. The average processing times for sub-grid and spot detections are shown in Table 2.

**Table 2:** Processing time of sub-grid and spot detection

	Sub-grid Detection	Spot Detection
SMD	379.1	10.8

GEO	384.7	9.2

DILN	62.3	3.8

Average processing times (in seconds) for detecting sub-grids within each cDNA microarray image and detecting spots within each detected sub-grid.

### Sub-grid and Spot Detection Accuracy

Table 3 shows the results of applying the proposed method, OMTG, for spot detection on the SMD dataset. With the proposed method, spot locations can be detected very efficiently with an average accuracy of 98.06% for this dataset. The same sets of experiments were repeated for the GEO dataset and the results are shown in Table 4. Again, the spot locations are detected very efficiently with an average accuracy of 99.26%. The experiments were repeated for the DILN dataset and the results are shown in Table 5. Although the sizes of the spots in each sub-grid are different in this dataset, the spot locations are detected very efficiently with an average accuracy of 97.95%. In most of the images, the performance of the method is more than 98% and incorrectly and marginally aligned rates are less than 1%. Only in a few images with noticeable noise and defects, the accuracy of the method is less than 98%, while incorrectly aligned rates increase to more than 2%. This shows the flexibility and power of the proposed method. For all the images, in the sub-grid detection phase, the incorrect and marginal gridding rates are both 0%, yielding an accuracy of 100%. This means the proposed method works perfectly in sub-grid detection for this case. One of the reasons for the lower accuracy in spot detection is that the distance between spots is smaller than the distance between sub-grids. In all three datasets, there are approximately eight pixels between spots, and approximately 30 pixels horizontally and 100 pixels vertically between sub-grids in the SMD dataset, 200 pixels in the GEO dataset and 25 pixels horizontally, and 200 pixels vertically in the DILN dataset. Another possible reason for this behavior is that the number of pixels in each sub-grid is far lower than that of a microarray image (around 1/50). Thus, the noise present in the image affects the spot detection phase much more than the sub-grid extraction stage. It is important to highlight, however, that because of the relatively large distance between sub-grids, the detection process is not affected by the presence of noise.

**Table 3:** Accuracy of the proposed method on the SMD dataset

	Sub-grid Detection			Spot Detection		
**Image**	**Incorrectly**	**Marginally**	**Perfectly**	**Incorrectly**	**Marginally**	**Perfectly**

AT-20385-CH1	0.0%	0.0%	100%	4.30%	0.46%	95.24%

AT-20385-CH2	0.0%	0.0%	100%	2.83%	0.09%	97.08%

AT-20387-CH1	0.0%	0.0%	100%	2.90%	0.14%	96.96%

AT-20387-CH2	0.0%	0.0%	100%	0.52%	0.11%	99.37%

AT-20391-CH1	0.0%	0.0%	100%	0.64%	0.17%	99.19%

AT-20391-CH2	0.0%	0.0%	100%	0.32%	0.26%	99.42%

AT-20392-CH1	0.0%	0.0%	100%	4.10%	0.33%	95.57%

AT-20392-CH2	0.0%	0.0%	100%	0.21%	0.25%	99.54%

AT-20395-CH1	0.0%	0.0%	100%	0.41%	0.12%	99.47%

AT-20395-CH2	0.0%	0.0%	100%	0.98%	0.31%	98.71%

Accuracy of detected sub-grids and spots for each image of the SMD dataset and the corresponding incorrectly, marginally and perfectly aligned rates.

**Table 4:** Accuracy of the proposed method on the GEO dataset

	Sub-grid Detection			Spot Detection		
**Image**	**Incorrectly**	**Marginally**	**Perfectly**	**Incorrectly**	**Marginally**	**Perfectly**

GSM15898-CH1	0.0%	0.0%	100%	0.58%	0.16%	99.26%

GSM15899-CH2	0.0%	0.0%	100%	1.00%	0.21%	98.79%

GSM16101-CH1	0.0%	0.0%	100%	0.00%	0.32%	99.68%

GSM16101-CH2	0.0%	0.0%	100%	1.57%	0.06%	98.37%

GSM16389-CH1	0.0%	0.0%	100%	0.79%	0.12%	99.09%

GSM16389-CH2	0.0%	0.0%	100%	0.57%	0.04%	99.39%

GSM16391-CH1	0.0%	0.0%	100%	0.00%	0.24%	99.76%

GSM16391-CH2	0.0%	0.0%	100%	0.14%	0.13%	99.73%

Accuracy of detected sub-grids and spots for each image of the GEO dataset and the corresponding incorrectly, marginally and perfectly aligned rates.

**Table 5:** Accuracy of the proposed method on the DILN dataset

	Sub-grid Detection			Spot Detection		
**Image**	**Incorrectly**	**Marginally**	**Perfectly**	**Incorrectly**	**Marginally**	**Perfectly**

Diln4-3.3942.01A	0.0%	0.0%	100%	2.23%	0.05%	97.72%

Diln4-3.3942.01B	0.0%	0.0%	100%	1.71%	0.11%	98.18%

Accuracy of detected sub-grids and spots for each image of the DILN dataset and the corresponding incorrectly, marginally and perfectly aligned rates.

Additionally, to evaluate the effectiveness of the refinement procedure, we tested the accuracy of the proposed method with and without applying the refinement procedure. The results are shown in Table 6. For simplicity, we only include those images in which there is a change in accuracy. We observe that applying the refinement procedure slightly improve the efficiency of the method in all the images in the table.

**Table 6:** Effectiveness of the refinement procedure

	Without Refinement Procedure			With Refinement Procedure		
**Image**	**Incorrectly**	**Marginally**	**Perfectly**	**Incorrectly**	**Marginally**	**Perfectly**

AT-20385-CH1	4.73%	0.79%	94.48%	4.30%	0.46%	95.24%

AT-20387-CH2	0.93%	0.54%	98.53%	0.52%	0.11%	99.37%

AT-20391-CH2	0.71%	0.58%	98.71%	0.32%	0.26%	99.42%

AT-20395-CH2	1.37%	0.76%	97.87%	0.98%	0.31%	98.71%

GSM16101-CH2	2.13%	0.21%	97.66%	1.57%	0.06%	98.37%

GSM16389-CH1	0.93%	0.19%	98.88%	0.79%	0.12%	99.09%

GSM16391-CH2	0.47%	0.26%	99.27%	0.14%	0.13%	99.73%

The accuracy of the proposed method with and without using the refinement procedure in the spot detection phase. Only images with changes in accuracy are listed.

**Figure 1:**
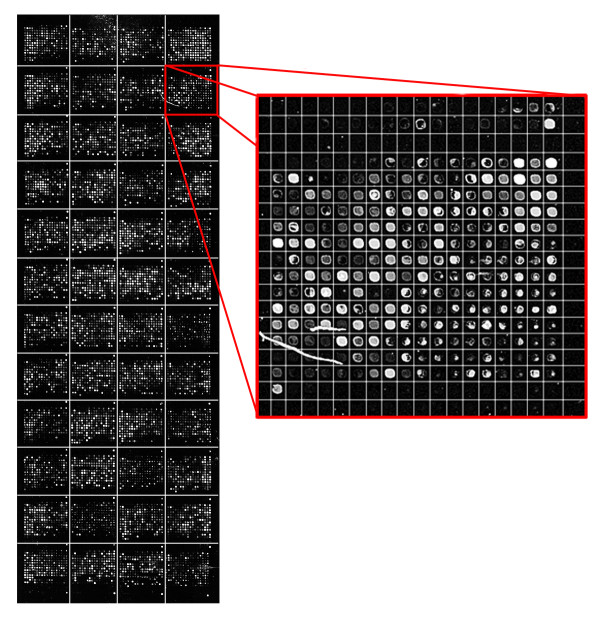
**Sub-grid and spot detection in one of the SMD dataset images**. Detected sub-grids in AT-20387-ch2 (left), and detected spots in one of the sub-grids (right).

**Figure 2:**
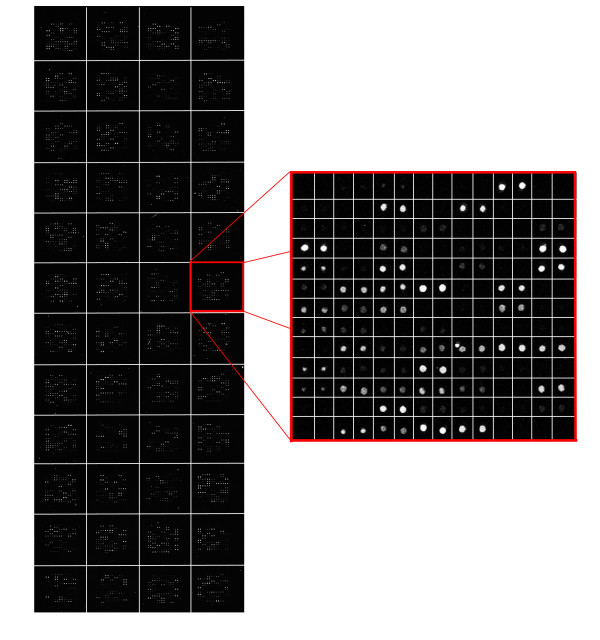
**Sub-grid and spot detection in one of the GEO dataset images**. Detected sub-grids in GSM16101-ch1 (left), and detected spots in one of the sub-grids (right).

**Figure 3:**
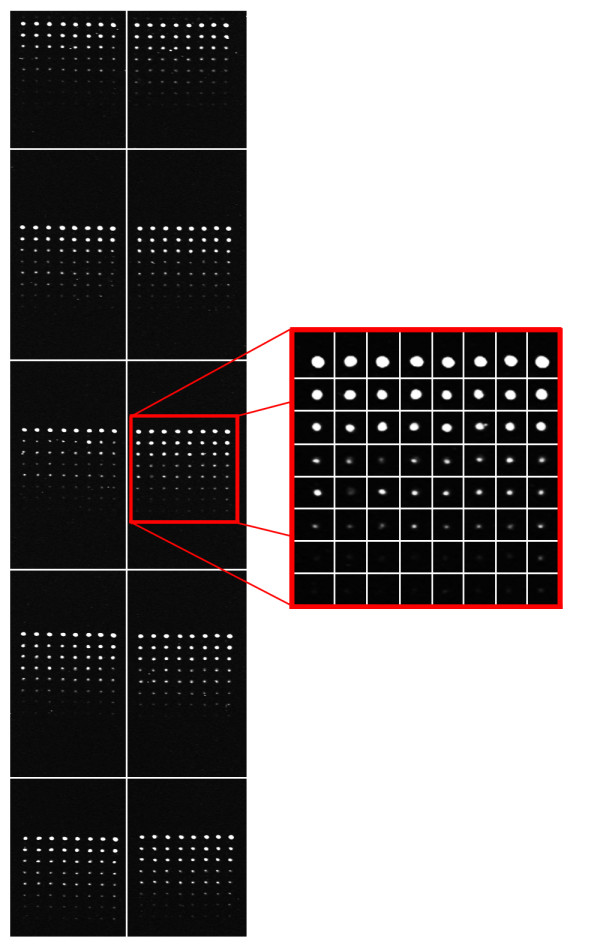
**Sub-grid and spot detection in one of the DILN dataset images**. Detected sub-grids in Diln4-3.3942B (left) and detected spots in one of the sub-grids (right).

To analyze the results from a different perspective, we have also performed a visual analysis. Figure 1 shows the detected sub-grids in the AT-20387-ch2 image (left) and the detected spots in one of the sub-grids (right). Also, Figure 2 shows the sub-grids detected in the GSM16101-ch1 image (left) and the detected spots in one of the sub-grids (right), while Figure 3 shows the sub-grids detected in the Diln4-3.3942B image (left) and the detected spots in one of the sub-grids (right). As shown in the all three figures, the proposed method finely detects the sub-grid locations first, and in the next stage, each sub-grid is divided precisely into the corresponding spots with the same method. The robustness of OMTG is so high that spots in sub-grids can be detected very well even in noisy conditions, such as those observable in the selected sub-grid in Figure 1. The ability to detect sub-grids and spots in different microarray images with different resolutions and spacing is another important feature of the proposed method.

**Figure 4:**
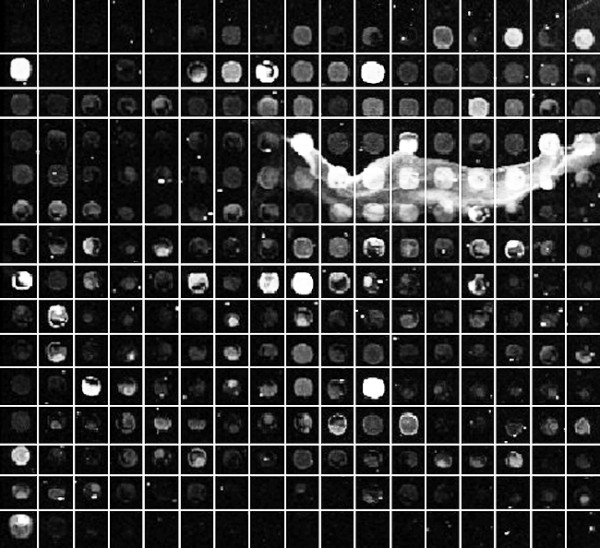
**Failure to detect the regions of some of the spots**. Failure to detect some spot regions due to the extremely contaminated images with artifacts in the sub-grid located in the first row and fourth column of AT-20392-ch1 from the SMD dataset.

As mentioned earlier, deformations, noise and artifacts can affect the accuracy of the proposed method. Figure 4 shows an example in which the proposed method fails to detect some spot regions due to the extremely contaminated regions with noise and artifacts. In this particular sub-grid, noisy regions tend to be confused with spots. Also, most spots have low intensities that are confused with the background. After testing other methods on this image, we observed that they also fail to detect the correct gridding in these regions.

**Figure 5:**
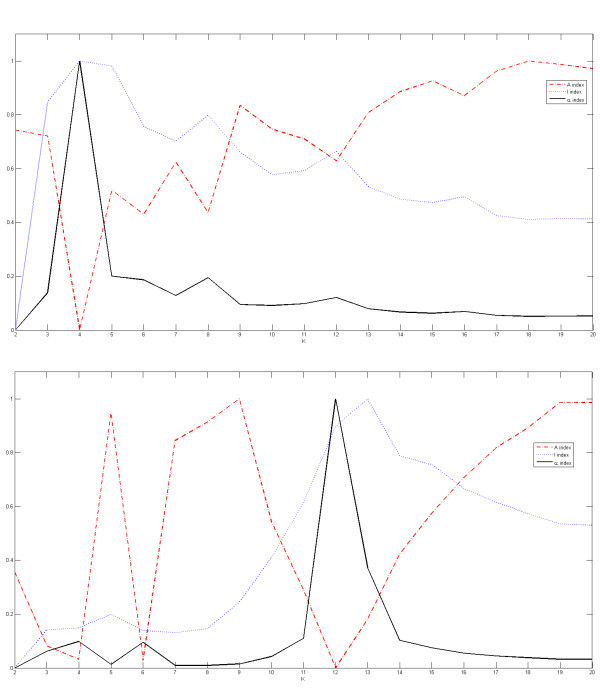
**Detection of the number of sub-grids in one of images of SMD**. Plots of the index functions for AT-20387-ch2: (top) the values of the *I*, *A *and *α *indices for horizontal separating lines, and (bottom) the values of the *I*, *A *and *α *indices for vertical separating lines.

**Figure 6:**
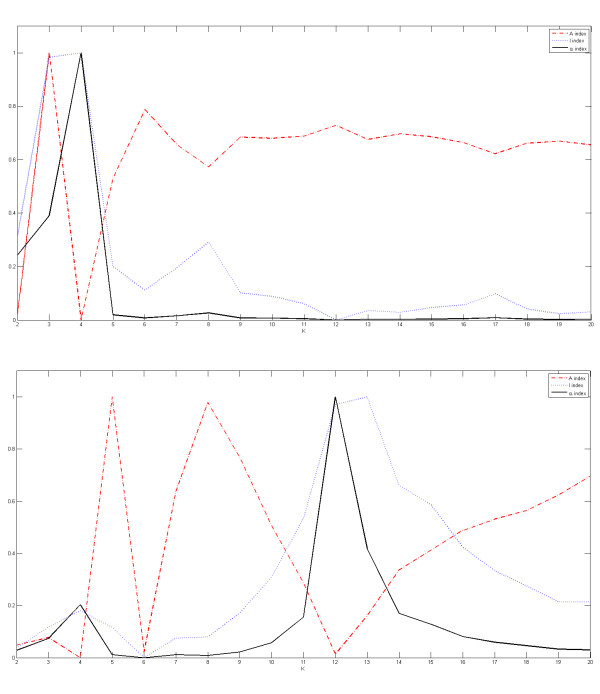
**Detection of the number of sub-grids in one of images of GEO**. Plots of the index functions for the GSM16101-ch1: (top) the values of the *I*, *A *and *α *indices for horizontal separating lines, and (bottom) the values of the *I*, *A *and *α *indices for vertical separating lines.

**Figure 7:**
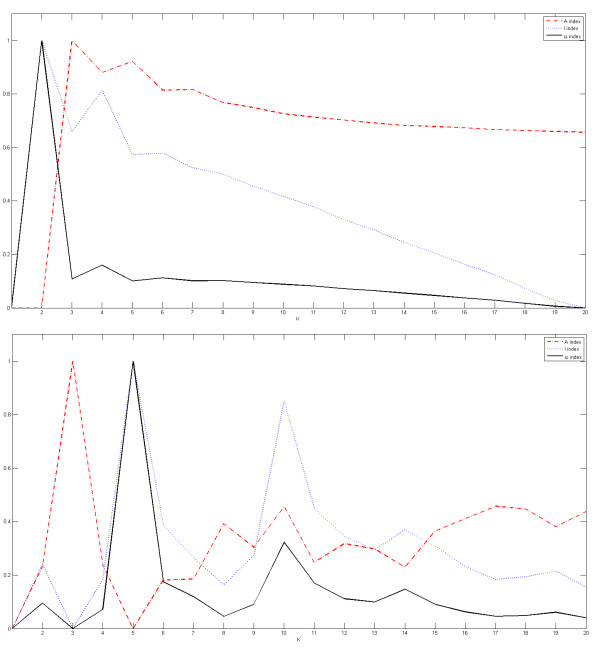
**Detection of the number of sub-grids for one of images of the DILN dataset**. Plots of the index functions for the Diln4-3.3942B: (top) the values of the *I*, *A *and *α *indices for horizontal separating lines, and (bottom) the values of the *I*, *A *and *α *indices for vertical separating lines.

To further analyze the efficiency of the proposed method to automatically detect the correct number of spots and sub-grids, we show in Figures 5, 6 and 7 the plots for the indices of validity against the number of sub-grids for AT-20387-ch2, GSM16101-ch1 and Diln4-3.3942B respectively. The plots on top of the figures represent the values of the index functions (*y *axis) for detecting the horizontal lines for the *I*, *A *and *α *indices respectively, while the plots of the indices for the vertical separating lines are shown at the bottom of the figures. We observe that it would be rather difficult to find the correct number of sub-grids using the *I *index or the *A *index, while the *α *index clearly reveals the correct number of horizontal and vertical sub-grids by producing an almost flat curve with pronounced peaks at 4 and 12 respectively for SMD and GEO images, and pronounced peaks at 2 and 5 respectively for DILN images. For example, it is clearly observable at the bottom plots in Figures 5 and 6 that the *I *index misses the correct number of sub-grids, 12, by showing a higher peak at 13, while the α index finds the correct number of vertical sub-grids accurately.

### Rotation Adjustment Accuracy

**Figure 8:**
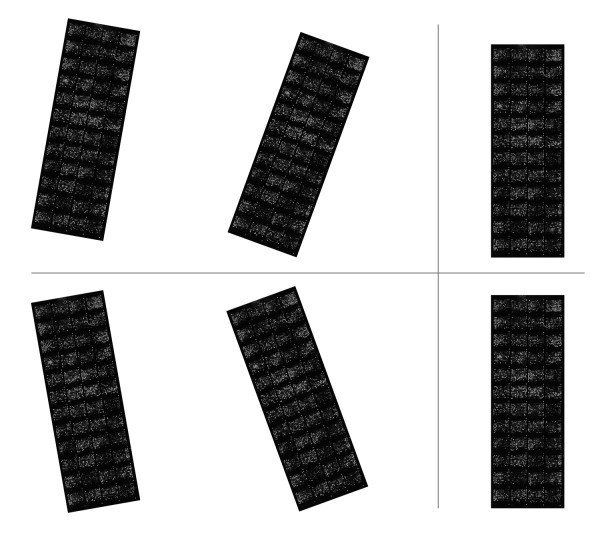
**Rotation adjustment**. Rotation adjustment of AT-20387-ch2. Four different rotations from -20 to 20 degrees with steps of 10 degrees (left), and the adjusted image after applying the Radon transform (right).

To test the effect of the Radon transform we rotate two of the images 5,10,15,20 and 25 degrees in both clockwise and counter-clockwise directions. Figure 8 shows the images rotated by -20, -10, 10 and 20 degrees (left) and the result of the adjustment after applying the Radon transform (right). Also, Table 7 shows the accuracy of the proposed method on two of the rotated images. In all cases, the adjustment method works accurately and corrects the rotations in both directions. Moreover, as shown in Table 7, the accuracy of the method remains nearly constant for all cases regardless of the degree of rotation. This shows the effectiveness and robustness of the proposed method in significantly rotated images.

**Table 7:** Accuracy of the proposed method on the rotated images

	AT-20395-CH1			GSM16391-CH2		
**Rotation**	**Incorrectly**	**Marginally**	**Perfectly**	**Incorrectly**	**Marginally**	**Perfectly**

none	0.41%	0.12%	99.47%	0.14%	0.13%	99.73%

5°	0.41%	0.12%	99.47%	0.14%	0.13%	99.73%

10°	0.43%	0.12%	99.45%	0.15%	0.14%	99.71%

15°	0.41%	0.13%	99.46%	0.14%	0.13%	99.73%

20°	0.42%	0.13%	99.45%	0.15%	0.14%	99.71%

25°	0.42%	0.15%	99.43%	0.14%	0.15%	99.71%

-5°	0.41%	0.12%	99.47%	0.14%	0.13%	99.73%

-10°	0.41%	0.12%	99.47%	0.14%	0.13%	99.73%

-15°	0.42%	0.13%	99.45%	0.14%	0.14%	99.72%

-20°	0.42%	0.14%	99.44%	0.15%	0.13%	99.72%

-25°	0.42%	0.16%	99.42%	0.14%	0.15%	99.71%

Accuracy of detected spots for different rotations of AT-20395-CH1 and GSM16391-CH2, and the corresponding incorrectly, marginally and perfectly aligned rates.

### Comparison with other methods

A conceptual comparison between the proposed method, OMTG, and other microarray image griding methods based on their features is shown in Table 8. The methods included in the comparison are the following: (i) Radon transform sub-gridding (RTSG) [12], (ii) Bayesian simulated annealing gridding (BSAG) [3], (iii) genetic-algorithm-based gridding (GABG) [15], (iv) hill-climbing gridding (HCG) [5], (v) maximum margin microarray gridding (*M*^3^*G*) [16], and the proposed method, OMTG. As shown in the table, as opposed to other methods, OMTG does not need any number-based parameter, and hence making it much more powerful than the previous ones. One could argue, however, that the index or thresholding criterion can be considered as a "parameter". We have "fixed" these two on the *α index *and the *between class *criterion, and experimentally shown the efficiency of OMTG on various cDNA microarray images with different configurations.

**Table 8:** Conceptual comparison of different methods

Method	Parameters	Sub-grid Detection	Spot Detection	Automatic Detection No. of Spots	Rotation
RTSG	*n*: Number of sub-grids	√	×	×	√

BSAG	*α *,*β*: Parameters for balancing prior and posterior probability rates	×	√	√	√

GABG	*μ*, *c *:Mutation and Crossover rate, *p_max_*: probability of maximum thresh-old, *p_low_*: probability of minimum threshold, *f_max _*: percentage of line with low probability to be a part of grid, *T_p_*: Refinement threshold	√	√	√	√

HCG	*λ *, *σ*: Distribution parameters	×	√	√	×

*M *^3^*G*	*c*: Cost parameter	×	√	√	√

OMTG	None	√	√	√	√

Conceptual comparison of our proposed method with other recently proposed methods based on the required number and type of input parameters and features.

An experimental comparison of the proposed method with GABG and HCG is shown in Table 9. As opposed to the proposed method that needs no parameters, GABG needs to set several parameters such as the mutation rate, *μ*, the crossover rate, *c*, the maximum threshold probability, *p_max_*, the minimum threshold probability, *p_low_*, the percentage of lines with low probability to be a part of the grid, *f_max _*and the refinement threshold, *T_p_*. Also, HCG needs to set some parameters such as *λ *and *σ*. As shown in the table, the accuracy of our method is much higher than GABG and HCG. Since GABG and HCG use several parameters, to obtain good results for the SMD, GEO and DILN datasets, all the parameters must be set manually and separately for each dataset. If the same parameters for one of datasets were used for the others, unpredictable and poor results would be obtained - the accuracy of both methods could decrease to as low as 50%. This makes these methods fully dependent on the parameters, which have to be set manually and for specific datasets. The proposed method, however, does not need any parameter at all, and works exceptionally well in different kinds of images with different resolutions and noisy conditions.

**Table 9:** Comparison of the proposed method with GABG and HCG

Dataset	Method	Incorrectly	Marginally	Perfectly
SMD	OMTG	1.72%	0.22%	98.06%
	
	GABG	5.37%	0.51%	94.12%
	
	HCG	2.12%	1.23%	96.65%

GEO	OMTG	0.58%	0.16%	99.26%
	
	GABG	4.49%	0.32%	95.19%
	
	HCG	2.55%	0.74%	96.71%

DILN	OMTG	1.97%	0.08%	97.95%
	
	GABG	4.35%	0.34%	95.31%
	
	HCG	3.78%	0.65%	95.57%

The results of the comparison between the proposed method (OMTG) and the GABG and HCG methods proposed in [5] and [15] respectively.

### Biological Analysis

**Figure 9:**
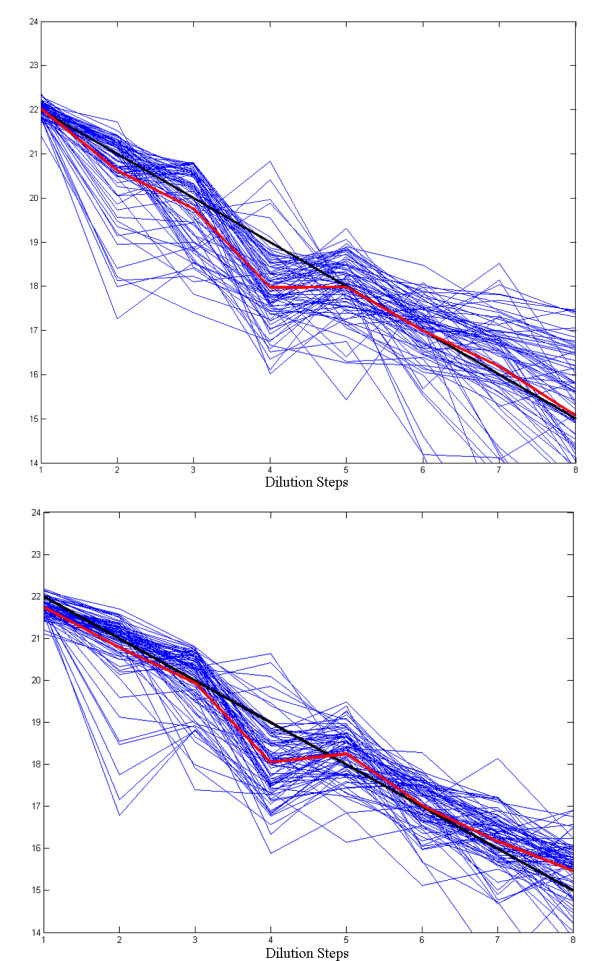
**Relationship between logs of spot volumes to the dilution steps**. The logs of spot volumes that correspond to the dilution steps in Diln4-3.3942.01A (top) and Diln4-3.3942.01B (bottom). The red lines show the average of logs of spot volumes in different dilution steps. The black line corresponds to the reference line with slope equal to -1.

**Figure 10:**
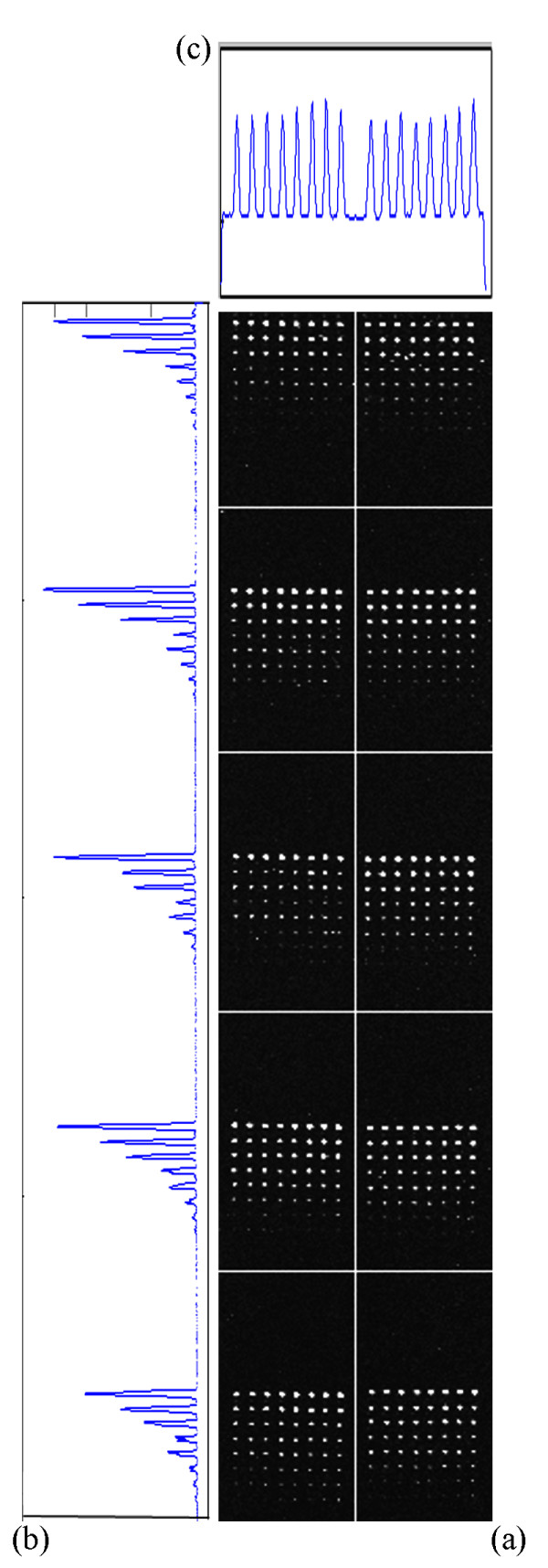
**Detected sub-grids and the corresponding horizontal and vertical histogram**. (a) detected sub-grids in Diln4-3.3942.01A, (b) vertical histogram (c) horizontal histogram.

In order to assess the proposed method on its suitability to perform in accordance with the biological problem, we analyze the quantification results and their relationships with the dilution experiment on the DILN dataset. To compute the volume intensity of each spot, first, we use *Sobel *method to detect the edge of each spot and then the region within the edge is defined as the primary region of each spot. In the next step, a set of morphological dilation and erosion operators are used to decrease the noise and artifacts in the region identified for each spot. Finally, the summation of all pixel intensities in the spot are used as the level of expression of the gene associated with that spot; this summation represents the *volume *of the spot. Table 10 shows the volume intensity of each dilution step for images A and B respectively. As shown in the table, the proposed method estimates the average intensities of dilution steps very well with near linear decreasing steps. Also, Figure 9 shows log-plots of the dilution steps for all 80 cases and the mean of them with a red line. The reference line with slope -1 is also shown in black. As shown in this figure, in most parts of the dilution experiment, the estimated intensities of each case follow a linear relationship. In step 4 of the dilution steps, there is an irregularity in the linearity of the red curve as shown in Table 10 and Figure 9. The reason for this irregularity is that, in some sub-grids of Diln4-3.3942.01A and Diln4-3.3942.01B, the intensities of the spots in step 4 are smaller than those of step 5. One example of this can be seen in the third and last rows of the sub-grids in Figure 10. As shown in Figure 10(b), this decrease in the intensity of the spots causes a slight nonlinearity in step 4 of the dilution steps. In general, we observe that the proposed method is able to capture the nonlinear relationships present in the dilution experiments. This is observable in the log-plots of Figure 9, as the black line follows the array of logs of spot volumes.

**Table 10:** Results on dilution experiments

Dilution steps	Diln4-3.3942.01A	Diln4-3.3942.01B
1	22.02	21.75

2	20.63	20.78

3	19.75	19.94

4	18.12	18.05

5	17.98	18.25

6	16.98	17.03

7	16.18	16.17

8	15.07	15.46

Logs of volume intensities of each dilution step for images A and B from the DILN dataset.

## Conclusions

A new method for separating sub-grids and spot centers in cDNA microarray images is proposed. The method performs four main steps involving the Radon transform for detecting rotations with respect to the *x *and *y *axes, the use of polynomial-time optimal multilevel thresholding to find the correct positions of the lines separating sub-grids and spots, a new index for detecting the correct number of sub-grids and spots and, finally, a refinement procedure to increase the accuracy of the detection.

The proposed method has been tested on real-life, high-resolution microarray images drawn from three sources, the SMD, GEO and DILN. The results show that (i) the rotations are effectively detected and corrected by affine transformations, (ii) the sub-grids are accurately detected in all cases, even in abnormal conditions such as extremely noisy areas present in the images, (iii) the spots in each sub-grid are accurately detected using the same method, (iv) using the refinement procedure increases the accuracy of the method, and (v) because of using an algorithm free of parameters, this method can be used for different microarray images in various situations, and also for images with various spot sizes and configurations effectively. The results have also been biologically validated on dilution experiments.

## Methods

A cDNA microarray image typically contains a number of sub-grids, and each sub-grid contains a number of spots arranged in rows and columns. The aim is to perform a two-stage process in such a way that the sub-grid locations are found in the first stage, and then spots locations within a sub-grid can be found in the second stage. Consider an image (matrix) *A *= {*a*_*i*,*j*_}, *i *= 1, ..., *n *and *j *= 1, ..., *m*, where *a_ij _*∈ ℤ^+^, and *A *is a sub-grid of a cDNA microarray image. The method is first applied to a microarray image that contains a template of rows and columns of sub-grids (usually, *a_ij _*is in the range [0..65,535] in a TIFF image). The aim of the first stage, sub-gridding, is to obtain vectors, **h **= [*h*_1_, ...*h*_*p*-1_]*^t ^*and **v **= [*v*_1_, ...*v*_*q*-1_]^*t*^, where *v_i _*∈[1, *m*], *h_j _*∈ [1, *n*] and *p *and *q *are the number of horizontal and vertical sub-grids respectively. These horizontal and vertical vectors are used to separate the sub-grids.

Ones the sub-grids are obtained, the gridding process, namely finding the locations of the spots in a sub-grid, can be defined analogously. The rectangular area between two adjacent horizontal vectors *h_j _*and *h*_*j*+1_, and two adjacent vertical vectors *v_i _*and *v*_*i*+1 _delimit the area corresponding to a spot (spot region). The aim of gridding is to find the corresponding spot locations given by the horizontal and vertical adjacent vectors. Post-processing or refinement allows us to find a spot region for each spot, which is enclosed by four lines.

To perform the gridding procedure our method may not need to know the number of sub-grids or spots. Although in many cases, based on the layout of the printer pins, the number of sub-grids or spots are known, due to misalignments, deformations, artifacts or noise during producing the microarray images, these numbers may not be accurate or unavailable. On the other hand, the optimal multi-level thresholding method needs the number of thresholds (sub-grids or spots) to be specified. Thus, we use an iterative approach to find the gridding for every possible number of thresholds, and then evaluate it with the proposed *α *index to find the best number of thresholds.

**Figure 11:**
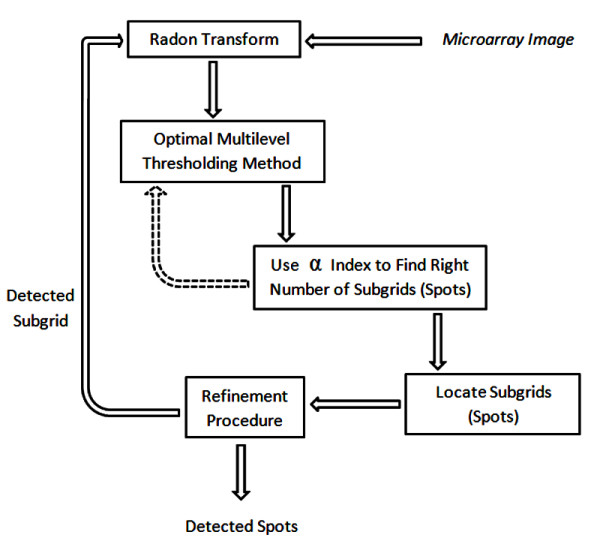
**The proposed method**. Schematic representation of the process for finding sub-grids (spots) in a cDNA microarray image.

The sub-grids in a microarray image are detected by applying the Radon transform as a pre-processing phase and then using optimal multilevel thresholding in the next stage. By combining the optimal multilevel thresholding method and the α index (12), the correct number of thresholds (sub-grids) can be found. Figure 11 depicts the process of finding the sub-grids in a microarray image and the spots in a sub-grid. The input to the Radon transform is a cDNA microarray image and the output of the whole process is the location (and partitioning) of the sub-grids. Analogously, the locations of the spots in each sub-grid are found by using optimal multilevel thresholding combined with the proposed α index to find the best number of rows and columns of spots. The input for this process is a sub-grid (already extracted from the sub-gridding step) and the output is the partitioning of the sub-grids into spots (spot regions).

### Rotation Adjustment

Rotations of the images are seen in two different directions, with respect to the *x *and *y *axes. To find two independent angles of rotation for an affine transformation, the Radon transform is applied. Given an image *A *= {*a_x,y_*}, the Radon transform performs the following transformation:(1)

where *p *is the slope and *t *its intercept. The rotation angle of the image with respect to the slope *p *is given by *ϕ *= arctan *p*. For the sake of the notation, *R*(*ϕ*, *t*) is used to denote the Radon transform of image *A*. Each rotation angle *ϕ *gives a different one-dimensional function, and the aim is to obtain the angle that gives the best alignment with the lines. This will occur when the lines are *parallel *to the *y*-axis. The best alignment will occur at the angle *ϕ_min _*that minimizes the *entropy *as follows [1]:(2)

*R*(*ϕ*, *t*) is normalized into *R'*(*ϕ*, *t*), such that Σ_*t *_*R'*(*ϕ*, *t*) = 1. The positions of the pixels in the new image,[*uv*], are obtained as follows:(3)

where  and  are the best angles of rotation found by the Radon transform.

### Optimal Multilevel Thresholding

**Figure 12:**
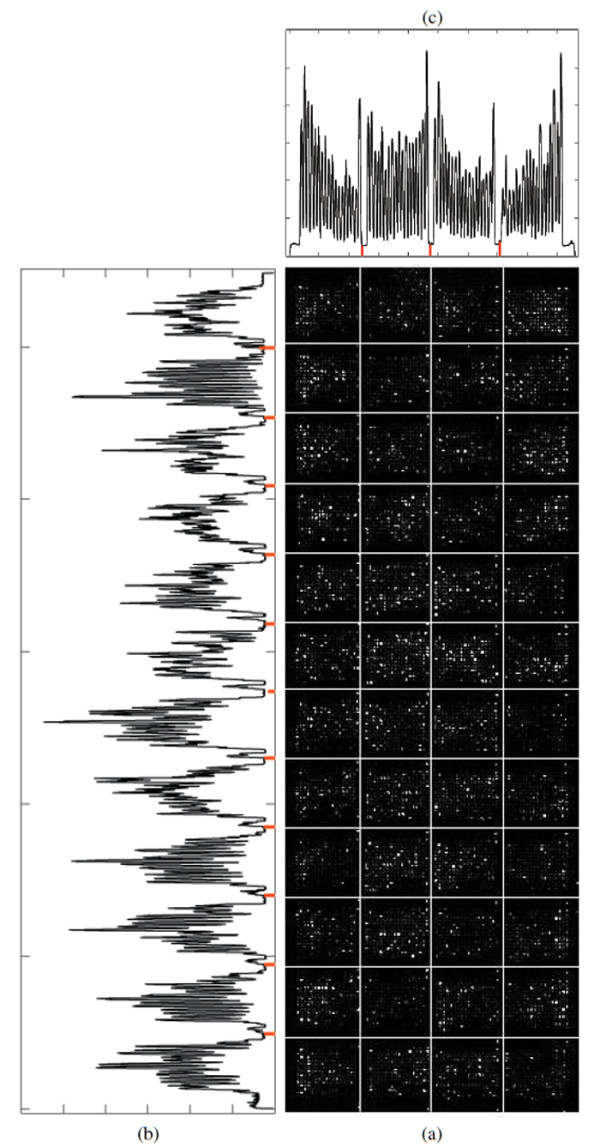
**Sub-grid detection in a microarray image from the SMD dataset**. (a) detected sub-grids in AT-20387-ch2 from the SMD dataset, (b) horizontal histogram and detected valleys corresponding to horizontal lines, (c) vertical histogram and detected valleys corresponding to vertical lines.

**Figure 13:**
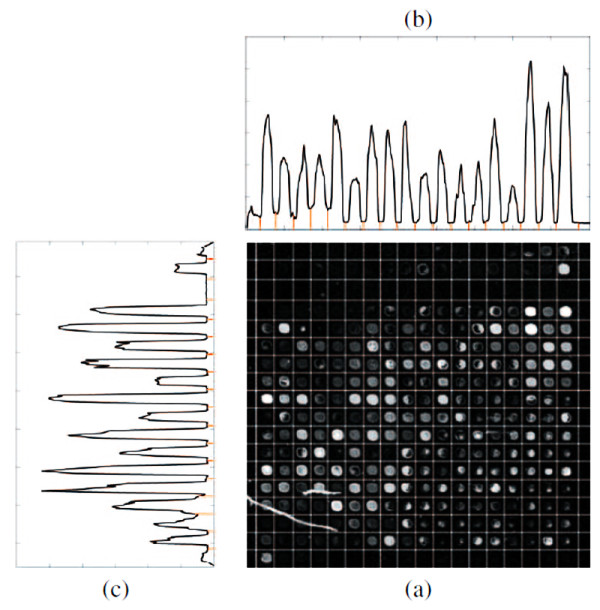
**Spot detection in a sub-grid from AT-20387-ch2**. (a) detected spots in one of the sub-grids in AT-20387-ch2, (b) horizontal histogram and detected valleys corresponding to horizontal lines, (c) vertical histogram and detected valleys corresponding to vertical lines.

Image thresholding is one of the most widely-used techniques that has many applications in image processing, including segmentation, classification and object recognition. Given a sub-grid, we compute the row or column sums of pixel intensities, obtaining a discrete one dimensional function, where the domain is given by the positions of the rows/columns of pixels. In this work, that function is considered as a histogram in which each bin represents one column (or row respectively), and the row or column sum of intensities corresponds to the frequency of that bin. We use the terms "histogram" or "sum" indistinctly. The frequencies are then normalized in order to be considered as probabilities of the corresponding bins. Figure 12 depicts a typical cDNA microarray image (AT-20387-ch2) that contains 12 × 4 sub-grids, along with the corresponding row or column sums. Also, Figure 13 depicts one of its sub-grids along with the corresponding row and column sums. Each row or column sum is then processed (see below) to obtain the optimal thresholding that will determine the locations of the sub-grids (spots).

Although various parametric and non-parametric thresholding methods and criteria have been proposed, the three most important streams are Otsu's method, which aims to maximize the separability of the classes measured by means of the sum of between-class variances [19], the one that uses information theoretic measures in order to maximize the separability of the classes [20], and the minimum error criterion [21]. In this work, we use the between-class variance criterion [19].

Consider a histogram *H*, an ordered set {1, 2, ..., *n *- 1, *n*}, where the *i*th value corresponds to the *i*th bin and has a probability, *p_i_*. Given an image, *A *= {*a_i,j_*}, as discussed earlier, *H *can be obtained by means of the horizontal (vertical) sum as follows: . We also consider a threshold set *T*, defined as an ordered set *T *= {*t*_0_, *t*_1_, ..., *t_k_*, *t*_*k*+1_}, where 0 = *t*_0 _<*t*_1 _< ... <*t*_*k *_<*t*_*k*+1 _= *n *and *t_i _*∈ {0} ∪ *H*. The problem of multilevel thresholding consists of finding a threshold set, T *, in such a way that a function *f *: *H^k ^*× [0, 1] *^n ^*→ *R*^+ ^is maximized/minimized. Using this threshold set, *H *is divided into *k *+ 1 classes: ζ_1 _= {1, 2, ..., *t*_1_}, ζ_2 _= {*t*_1 _+ 1, *t*_1 _+ 2, ..., *t*_2_}, ..., ζ*_k _*= {*t*_*k*-1 _+ 1, *t*_*k*-1 _+ 2, ..., *t*_*k*_}, ζ_*k*+1 _= {*t*_*k *_+ 1, *t*_*k *_+ 2, ..., *n*}. The between class variance criterion is given by:(4)

where , 

We use the dynamic programming algorithm for *optimal *multilevel thresholding proposed in [22], which is an extension for irregularly sampled histograms. To implement the between-class variance criterion, Ψ_BC_(*T*) is expressed as follows: , where . We consider the temporary variables *a *and *b *,which are computed as follows:(5)(6)

Since from (5) and (6), a and b are known, then , for the next step, can be re-computed as follows in Θ(1) time:(7)(8)(9)

Full details of the algorithm, whose worst-case time complexity is *O*(*kn*^2^), can be found in [22].

### Automatic Detection of the Number of Sub-grids and Spots

Finding the correct number of sub-grids and spots in each sub-grid is one of the most challenging issues in sub-grid and spot detection. This stage is crucial in order to fully automate the whole process. Multi-level thresholding uses the number of sub-grids (spots) as a single parameter. Thus, we need to determine the correct number of sub-grids (spots) prior to using multi-level thresholding methods. For this, we resort on validity indices used for clustering. By analyzing the traditional indices for clustering validity and their suitability to be combined with our measure, we propose a new index of validity for this specific problem. From the different indices of validity for clustering (cf. [23,24]), we consider the *I *index as the basis of the proposed index. The *I *index is defined as follows:(10)

where , , *n *is the total number of points in the dataset (bins in the histogram), and *z_k _*is the center of the *k*th cluster. We also consider the average frequency value of the thresholds in a histogram, which is computed as follows:(11)

where *t_i _*is the *i*th threshold found by optimal multilevel thresholding and *p*(*t_i_*) is the corresponding probability value in the histogram.

The proposed index, *α*(*x*), is the result of a combination of *A*(*K*), (11) and the *I *index, (10), as follows:(12)

For maximizing *I*(*K*) and minimizing *A*(*K*), the value of *α*(*K*) must be maximized. Thus, the best number of thresholds K* based on the *α *index is given by:(13)

To find the best number of thresholds, K *, we perform an exhaustive search on all positive values of *K *from 1 to *δ *and find the value of *k *that maximizes the *α *index. In our experiment we set *δ *to  (cf. [25]).

### The Refinement Procedure

In some cases, the detected grid or sub-grid may not separate spots completely or may separate them marginally. In these cases, a refinement procedure can be used to boost the performance of method. For this, each horizontal or vertical line is replaced with a new line. Consider two horizontal lines *h_j _*and *h*_*j*+1 _where *j *∈[1, K* ] and a vertical line *v_i _*where *i *[1, K*], and *v_i _*is bounded between *h_j _*and *h*_*j*+1_. Given *A *= {*a_ij _*}, line *v_i _*can be moved to left and right in such way that  is minimized. In other words, the vertical line *v_i _*can be replaced with a new vertical line, *v_r_*, in such a way that:(14)

**Figure 14:**
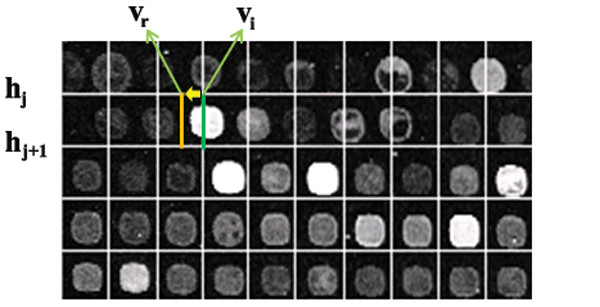
**The refinement procedure**. During the refinement procedure each line can be moved to left or right (for vertical lines) and up or down (for horizontal lines) to find the best location separating the spots. In this image, *v_i _*is the sub-line before using the refinement procedure and *v_r _*is the sub-line after adjusting it during refinement procedure.

Analogously, this procedure can be applied to each horizontal line. Figure 14 shows an example in which a vertical line is replaced by a new one during the refinement procedure. As shown in the figure, the vertical line *v_i _*is originally located in the wrong place and does not separate two adjacent spots correctly. By moving it to left and right, the new line *v_r _*is found in such way that those adjacent spots are separated correctly.

**Figure 15:**
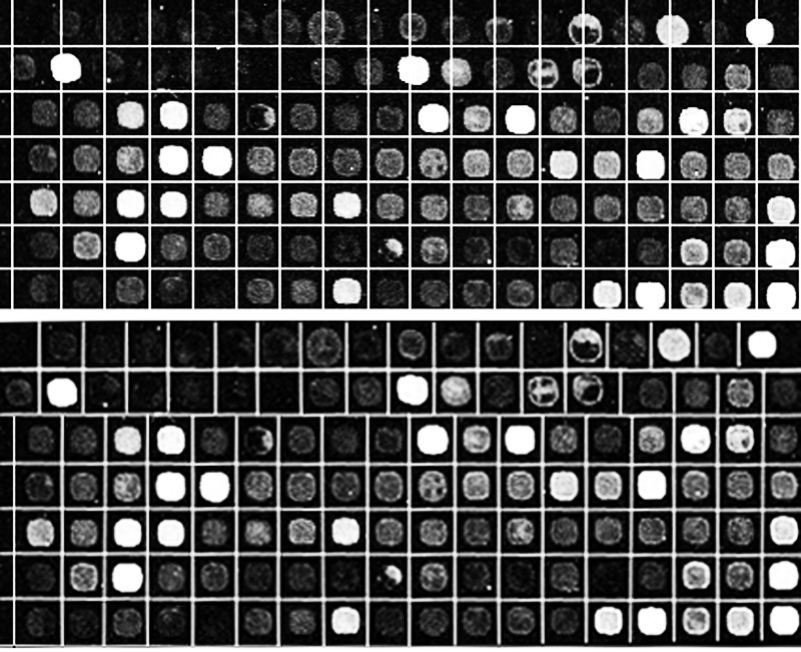
**Effect of the refinement procedure to increase the accuracy of the proposed method**. Detected spots in one of the sub-grids of AT-20387-ch1 from the SMD dataset before using the refinement procedure (top), and detected spots in the same part of the sub-grid after using the refinement procedure (bottom).

Figure 15 shows the detected spots in one of the sub-grids of 20387-ch2 of SMD before and after using the refinement procedure. It is clear that there are some misalignments in separating the adjacent spots in the top part of the sub-grid before using the refinement procedure. After the refinement, all the spots are separated precisely as shown in the figure.

## Authors' contributions

LR and IR conceived the gridding model, performed the data analysis, and elaborated the corresponding discussions. IR implemented the algorithms and conducted the experiments. Both authors have read and approved the final manuscript.
